# Immune checkpoint inhibitor-related cardiotoxicity: a cellular pharmacology perspective on susceptibility and resistance

**DOI:** 10.3389/fphar.2026.1845366

**Published:** 2026-06-11

**Authors:** Hui Fang, Bingjie Guo, Yajun Sun, Wujia Jin, Wenting Wang

**Affiliations:** 1 Department of Thoracic Oncology (II), Hangzhou Cancer Hospital, Hangzhou, China; 2 Department of Thoracic Oncology (I), Hangzhou Cancer Hospital, Hangzhou, China; 3 Department of Integrated Oncology (I), Hangzhou Cancer Hospital, Hangzhou, China

**Keywords:** cardiotoxicity, cellular pharmacology, immune checkpoint inhibitors, immune dysregulation, myocarditis

## Abstract

Immune checkpoint inhibitors (ICIs) have reshaped the treatment landscape of cancer, yet their clinical benefit is accompanied by a distinct spectrum of immune-related toxicities. Among these, cardiotoxicity remains uncommon but clinically consequential because it can evolve quickly, presents with marked heterogeneity, and may result in fulminant myocarditis, malignant arrhythmia, or hemodynamic collapse. Key mechanistic drivers include T-cell clonality directed against cardiac autoantigens (e.g., α-myosin heavy chain), cytokine amplification via JAK/STAT signaling, and innate immune recruitment. Recent work has shifted the field away from viewing ICI-related cardiotoxicity as a nonspecific inflammatory complication. Instead, available data support a model in which immune checkpoint blockade perturbs a broader tumor–host ecosystem and exposes organ-specific vulnerabilities within the heart. T-cell clonality, loss of peripheral tolerance, cytokine amplification, endothelial activation, stromal remodeling, and metabolic rewiring all appear to contribute, although their relative importance likely varies across patients. This review examines ICI-associated cardiotoxicity through the lens of cellular pharmacology. We focus on how checkpoint signaling sustains cardiac immune homeostasis, how susceptibility emerges from interactions between tumor-derived cues and host immune context, and why resistance to toxicity-directed therapy remains clinically relevant. We also discuss evolving biomarker strategies—including high-sensitivity troponins, cardiac MRI, and emerging immune-state markers—and mechanism-based interventions that may help reduce cardiac injury without fully negating antitumor efficacy. By framing cardiotoxicity as a context-dependent extension of systemic immune modulation rather than an isolated adverse event, we highlight unresolved questions that are central to the development of predictive biomarkers and more selective therapeutic strategies. Finally, we briefly note that structured nursing surveillance and multidisciplinary team coordination remain essential for translating mechanistic advances into improved bedside outcomes.

## Introduction

The rapid expansion of immune checkpoint inhibitors into routine oncology practice has changed expectations for durable cancer control across multiple tumor types. Antibodies targeting PD-1, PD-L1, and CTLA-4 have produced meaningful and often sustained benefit in melanoma, lung cancer, renal cell carcinoma, hepatocellular carcinoma, and several hematologic malignancies, while newer combinations that incorporate LAG-3 blockade continue to broaden the therapeutic repertoire (Ribas and Wolchok, 2018; Sharma and Allison, 2020; Hodi et al., 2010; Borghaei et al., 2015; Motzer et al., 2015; Robert, 2020; Rotte, 2019; Twomey and Zhang, 2021; Zhang et al., 2020a; Shi et al., 2019; Tawbi et al., 2022).

That therapeutic success comes with a persistent biological trade-off. The same signaling axes that tumors exploit to evade immune elimination also serve as core regulators of peripheral tolerance. Once those restraints are lifted, toxicities can emerge in virtually any organ system. Cardiac events are less common than dermatologic, gastrointestinal, or endocrine immune-related adverse events, but they carry disproportionate clinical weight because deterioration can be abrupt and mortality remains high, particularly in myocarditis (Postow et al., 2018; Martins et al., 2019; Brahmer et al., 2018; Wang et al., 2018; Khunger et al., 2017; Salem et al., 2018; Moslehi et al., 2018; Dolladille et al., 2021; Mahmood et al., 2018; Johnson et al., 2016). The reported incidence of ICI-related myocarditis ranges from 0.04% to 1.14% in clinical trial populations, although real-world registries suggest rates may be higher (Tang et al., 2025; Ozaki et al., 2025). Mortality for fulminant myocarditis remains 25%–50%, and combination ICI regimens (anti-CTLA-4 plus anti-PD-1) carry an approximately 5-fold higher risk of cardiotoxicity compared with monotherapy, with myocarditis incidence approaching 1.3% (Salem et al., 2018; Mahmood et al., 2018; Sharma et al., 2024).

A uniform explanation for ICI-associated cardiotoxicity has not emerged. Some patients present with isolated troponin elevation and limited symptoms, whereas others progress within days to heart block, ventricular dysfunction, or cardiogenic shock. The spectrum extends beyond myocarditis and includes pericardial disease, Takotsubo syndrome, conduction disturbances, arrhythmias, and acceleration of atherosclerotic inflammation (Escudier et al., 2017; Lyon et al., 2020; Palaskas et al., 2020; Altan et al., 2019; Mudra et al., 2018; Ederhy et al., 2019; Drobni et al., 2020; Drobni et al., 2023). Such variability argues against a single dominant pathway and instead points to differences in immune activation thresholds, myocardial susceptibility, and the broader tumor–host context in which toxicity develops.

This review considers ICI-related cardiotoxicity as a problem of cellular pharmacology. Rather than cataloguing every reported cardiac event, we focus on three linked questions: how checkpoint signaling maintains immune balance in cardiac tissue, what cellular and molecular processes shape susceptibility to injury, and why some patients respond poorly to toxicity-directed therapy. Framing the field in this way aligns cardiotoxicity with the larger discussion of sensitivity and resistance now central to cancer immunology (Axelrod et al., 2022; Munir et al., 2026; Salem et al., 2023). Specifically, we clarify that this framework differs from existing reviews by emphasizing how checkpoint signaling maintains cardiac immune homeostasis at the cellular level, how susceptibility emerges from interactions between specific cell populations (T cells, macrophages, fibroblasts, endothelial cells) and their signaling circuits, and how therapeutic resistance can be understood through mechanism-based pharmacological logic rather than empiric escalation. This distinguishes our review from purely clinical or pathological summaries by systematically linking each cardiotoxicity phenotype to its underlying cellular pharmacology (Table 1). We have also added a conceptual paragraph distinguishing our approach from translational reviews that focus primarily on biomarkers or clinical management algorithms.

**TABLE 1:** Representative preclinical models and key mechanistic insights in ICI-associated cardiotoxicity.

Model	Cardiac phenotype	Mechanistic insight	Key sources	Translational relevance and limitations
*Pdcd1* ^−/−^ *Ctla4* ^+/−^	Fulminant myocarditis with substantial penetrance	Supports a central role for autoreactive CD8^+^ T cells and provides a platform for mechanism-based intervention	Wei et al. (2021)	High relevance for testing mechanism-based interventions; limited by strain-specific penetrance and absence of tumor component
MRL-*Pdcd1* ^−/−^	Fatal autoimmune myocarditis	Demonstrates the requirement for PD-1-mediated tolerance in cardiac tissue	Nishimura et al. (2001), Wang et al. (2010),	Strong causal evidence for PD-1 dependence; lupus-prone background may not reflect typical cancer patient immunology
BALB/c-*Pdcd1* ^−/−^	Dilated cardiomyopathy	Links PD-1 deficiency to anti-troponin autoantibodies and humoral injury	Okazaki et al. (2003),	Links PD-1 loss to humoral injury; complete genetic knockout differs from pharmacological blockade
*Lag3* ^−/−^ *Pdcd1* ^−/−^	Arrhythmogenic myocarditis	Highlights the contribution of combinatorial checkpoint disruption and CXCR6+ T-cell localization	Munir et al. (2026), Gergely et al. (2024a),	Demonstrates combinatorial checkpoint disruption; does not model clinical dosing regimens
A/J + anti-PD-1	Drug-induced myocarditis without tumor	Suggests that pre-existing myosin-reactive T cells can become pathogenic after checkpoint blockade	Won et al. (2022),	Most closely mimics clinical drug administration; limited to a single strain with known cardiac susceptibility

## Literature search strategy

Literature for this review was identified through a comprehensive search of PubMed, Web of Science, and Embase (through March 2026) using the terms “immune checkpoint inhibitor” OR “ICI” combined with “cardiotoxicity” OR “myocarditis” OR “cardiac” combined with “mechanism” OR “pharmacology” OR “biomarker” OR “resistance.” We included peer-reviewed original research, systematic reviews, meta-analyses, and clinical guidelines published in English, with selective inclusion of case reports offering unique mechanistic insights. Studies published between 2018 and 2026 were prioritized to reflect recent developments, while seminal earlier works were retained. Two authors independently screened titles and abstracts, with disagreements resolved through discussion with the senior author.

Before reviewing the evidence, several key terms used throughout this review warrant concise definition. “Cardiac immune homeostasis” refers to the dynamic equilibrium between immune surveillance and tissue-protective tolerance in cardiac tissue, maintained through checkpoint pathways, regulatory T cells, and tissue-resident immune populations. “Tissue tolerance” describes the capacity of cardiac tissue to accommodate immune activity without sustaining clinically significant structural or functional injury, analogous to the concept of infection tolerance in host–pathogen biology. “Susceptibility” is used here to denote the conditional probability of developing cardiotoxicity given a specific combination of host immune architecture, tumor-derived factors, and treatment variables. These terms are distinguished from “vulnerability” (an inherent structural or functional predisposition) and “risk” (the overall likelihood of an adverse event estimated from population-level data).

## Checkpoint signaling and cardiac immune homeostasis

The heart is not immunologically inert. Cardiomyocytes, endothelial cells, and resident immune populations participate in a tightly regulated local environment in which checkpoint pathways help limit collateral damage from activated lymphocytes. PD-L1 is expressed in cardiac tissue and interacts with PD-1 on circulating or tissue-resident T cells to preserve local tolerance, while CTLA-4 constrains priming events upstream in secondary lymphoid organs (Grabie et al., 2019; Moslehi et al., 2021; Tarrio et al., 2012; Nishimura et al., 2001; Okazaki et al., 2003; Waterhouse et al., 1995; Sharpe and Pauken, 2018).

Evidence for this protective architecture predates the clinical era of ICIs. PD-1-deficient mice develop severe autoimmune cardiac phenotypes, including dilated cardiomyopathy and myocarditis, demonstrating that checkpoint pathways are integral to myocardial immune equilibrium rather than optional suppressive circuits (Nishimura et al., 2001; Okazaki et al., 2003). CTLA-4 loss is similarly catastrophic at the systemic level, and combined disruption of PD-1 and CTLA-4 produces fulminant inflammatory disease with a striking cardiac component in preclinical models (Waterhouse et al., 1995; Sharpe and Pauken, 2018; Tivol et al., 1995; Wei et al., 2021; Wang et al., 2023).

LAG-3 adds another layer of regulation. Its co-deletion with PD-1 yields arrhythmogenic myocarditis in mice and reinforces the broader idea that toxicity is shaped by the structure of checkpoint combinations rather than by checkpoint inhibition in the abstract (Munir et al., 2026; Gergely et al., 2024a). More recent data suggesting a role for PD-1/PD-L1 signaling in neonatal cardiac regeneration further underscore that these pathways influence cardiac biology beyond conventional immune suppression (Vargas Aguilar et al., 2024).

These observations matter pharmacologically. When checkpoint inhibitors are administered in patients, they do not simply intensify antitumor immunity. They also disturb a pre-existing network of tissue-protective signals in the heart. Whether this disturbance remains subclinical or progresses to overt injury likely depends on how systemic immune activation intersects with cardiac-specific tolerance mechanisms.

## Cellular mechanisms of ICI-associated cardiotoxicity

ICI-related cardiotoxicity is best understood as a multicellular process that emerges when systemic immune activation converges on a vulnerable cardiac microenvironment. Although individual studies emphasize different mechanisms, a common pattern can be recognized: enhanced T-cell effector activity, failure of tolerance, inflammatory recruitment, and feed-forward tissue injury (Axelrod et al., 2022; Moslehi et al., 2021; Wei et al., 2021; Champion and Stone, 2020; Ganatra and Neilan, 2018; Reuben et al., 2017; Bockstahler et al., 2020; Rubio-Infante et al., 2022; Gong et al., 2023).

However, ICI-associated cardiotoxicity encompasses mechanistically distinct phenotypes. To improve clarity and translational utility, we discuss the cellular mechanisms underlying each major phenotype separately below.

### Myocarditis: T-cell responses, clonality, and autoantigen recognition

The strongest mechanistic data continue to place T cells at the center of disease. Experimental work has shown that cardiac injury can be driven by T cells specific for α-myosin heavy chain, providing a concrete autoantigen model rather than a vague inflammatory hypothesis (Axelrod et al., 2022). This finding is important because it links checkpoint blockade to the expansion or release of autoreactive clones that are ordinarily kept in check by peripheral regulatory mechanisms.

Human studies add nuance rather than contradiction. Shared T-cell receptor clonotypes have been described across tumor, myocardium, and skeletal muscle in patients with severe ICI myocarditis, suggesting that cross-reactive or overlapping antigen recognition may sometimes bridge antitumor immunity and off-target tissue damage (Johnson et al., 2016; Reuben et al., 2017; Zhu et al., 2022). At the same time, preclinical evidence indicates that overt tumor cross-reactivity is not always required. In some settings, pre-existing autoreactive T cells appear sufficient once PD-1-mediated restraint is removed (Gergely et al., 2024b; Won et al., 2022).

This may help explain why susceptibility is uneven. The clinically relevant issue may not be whether autoreactive clones exist at all, but whether they are already antigen-experienced, metabolically competent, and positioned to expand after checkpoint blockade. From that perspective, cardiotoxicity becomes a problem of threshold biology: a latent autoreactive repertoire becomes pathogenic only when sufficient costimulatory and inflammatory conditions are met. Notably, the evidence supporting T-cell-mediated autoantigen recognition as a causal driver of myocarditis is strong: knockout mouse models (Pdcd1^−/−^) that develop fatal myocarditis (Nishimura et al., 2001; Wang et al., 2010) and adoptive transfer experiments with α-myosin heavy chain-specific T cells (Axelrod et al., 2022) provide direct experimental proof of causality. By contrast, the association between shared TCR clonotypes across tumor and myocardium in human patients represents strong associative evidence that has not yet been confirmed by interventional studies. Observations linking HLA background, clonal hematopoiesis, and overlap syndromes to cardiotoxicity risk remain correlative and require prospective validation.

### Myocarditis: macrophages, stromal cells, and innate amplification

T cells do not injure the heart in isolation. Histopathology from both human and murine myocarditis shows that macrophages are abundant within the inflammatory infiltrate and likely contribute to amplification rather than initiation alone (Ganatra and Neilan, 2018; Rubio-Infante et al., 2022; Gong et al., 2023; Lehmann et al., 2021). Activated T cells release interferon-γ and TNF-α, which reinforce macrophage activation and can promote further chemokine production, thereby sustaining immune-cell recruitment into myocardial tissue.

Innate and stromal compartments add additional complexity. Inflammasome signaling, type I interferon pathways, and fibroblast-derived mediators have each been implicated in maintaining an inflammatory niche once tissue injury begins (Wu et al., 2023; Varricchi et al., 2017; Michel et al., 2022; Horiguchi et al., 2023). Endothelial cells and fibroblasts may alter adhesion molecule expression and chemokine gradients, effectively converting the injured heart into a site that is more permissive for ongoing leukocyte entry.

**FIGURE 1:**
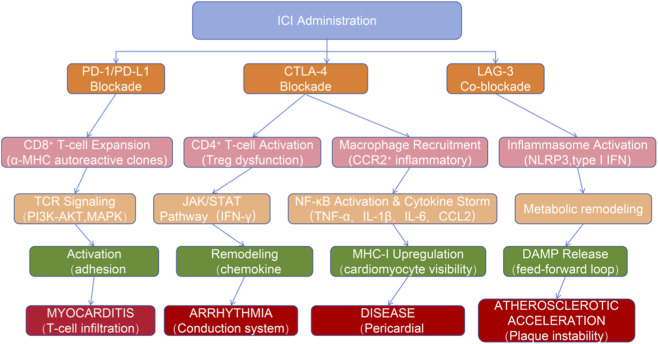
Cellular and molecular mechanisms underlying immune checkpoint inhibitor-associated cardiotoxicity.

This broader view is pharmacologically relevant because it implies that toxicity may persist even after the initiating T-cell burst has occurred. Once a self-reinforcing inflammatory microenvironment is established, intervention may need to target several nodes at once rather than relying on generalized corticosteroid therapy alone. These interacting cellular and molecular processes are summarized schematically in Figure 1, which highlights how checkpoint blockade perturbs cardiac immune homeostasis and amplifies tissue injury through coordinated T-cell, myeloid, stromal, and signaling circuits.

### Arrhythmias and conduction disturbances

Cardiac arrhythmias, including atrial fibrillation, ventricular tachycardia, and high-degree atrioventricular block, are the second most commonly reported cardiac events in ICI-treated patients. The underlying mechanisms appear to be distinct from those driving myocarditis. Inflammatory infiltration of the cardiac conduction system by activated lymphocytes and macrophages can directly disrupt electrical impulse propagation, particularly at the atrioventricular node and the His–Purkinje system. In addition, pro-inflammatory cytokines such as TNF-α, IL-1β, and IL-6 can modulate ion channel expression and function in cardiomyocytes, altering action potential duration and repolarization kinetics. Autonomic dysfunction triggered by systemic inflammation may further contribute to arrhythmogenesis. Unlike myocarditis, which has strong causal evidence from knockout mouse models, the mechanistic understanding of ICI-associated arrhythmias derives primarily from clinical case series and extrapolation from non-ICI inflammatory arrhythmia models. Prospective studies with systematic electrocardiographic monitoring are needed to clarify the incidence, timing, and mechanistic basis of these events (Escudier et al., 2017; Lyon et al., 2018; Palaskas et al., 2020; Altan et al., 2019; Herrmann, 2020).

### Pericardial disease

Pericarditis and pericardial effusion represent a distinct ICI-associated cardiotoxicity phenotype. In a large retrospective cohort of 88,928 ICI-treated patients, pericardial effusion occurred in 4.71% and pericarditis in 0.22%, making pericardial effusion among the most common cardiovascular events (Ozaki et al., 2025). The pathophysiology appears to involve immune-mediated inflammation of the pericardial mesothelium, with T-cell and macrophage infiltration of the pericardium rather than the myocardium. Pericardial involvement may occur in isolation or concurrently with myocarditis, and the degree of overlap has implications for both severity assessment and therapeutic approach. Patients with isolated pericardial disease generally carry a more favorable prognosis than those with concomitant myocarditis, though cardiac tamponade remains a life-threatening complication. The mechanistic distinction between pericardial and myocardial involvement is clinically relevant because pericarditis may respond to anti-inflammatory agents (colchicine, NSAIDs) that would be insufficient for myocarditis, while high-dose corticosteroids remain the first-line approach for both (Mudra et al., 2024; Ederhy et al., 2019; Drobni et al., 2020; Ozaki et al., 2025).

### Atherosclerotic acceleration

Accelerated atherosclerosis represents an increasingly recognized but mechanistically distinct cardiovascular consequence of ICI therapy. Checkpoint blockade can activate macrophages and T cells residing within coronary arterial plaques, intensifying local vascular inflammation and promoting plaque instability. PD-1 and CTLA-4 signaling normally restrain the activity of pro-atherogenic T cells in the arterial wall; removal of these restraints may shift the balance toward plaque rupture and acute coronary events. Large population-based studies have demonstrated that ICI-treated patients have an elevated risk of myocardial infarction and cerebrovascular events compared with matched cancer patients not receiving ICIs (Laenens et al., 2022; D'Souza et al., 2021). The evidence supporting this mechanism derives primarily from retrospective cohort analyses and epidemiological database studies rather than direct experimental models of ICI-induced plaque rupture. Unlike myocarditis, which can present acutely within weeks of ICI initiation, atherosclerotic events may develop over a more protracted time course, raising questions about the duration and nature of cardiovascular surveillance needed during and after ICI therapy. Emerging data suggest that statins and anti-inflammatory agents such as colchicine may have a role in mitigating ICI-associated vascular inflammation, though prospective trials are needed (Drobni et al., 2023; Laenens et al., 2022; D'Souza et al., 2021; Herrmann, 2020).

## Signaling and metabolic circuits shaping injury

Checkpoint blockade alters intracellular signaling in ways that are predictable in principle but variable in consequence. PD-1 engagement normally recruits phosphatase machinery that dampens proximal T-cell receptor signaling and restrains downstream PI3K–AKT and MAPK pathways. Blocking PD-1 therefore enhances proliferative and effector programs, while CTLA-4 blockade augments costimulatory signaling at the level of T-cell priming (Keir et al., 2008; Patsoukis et al., 2020).

Those changes become clinically meaningful when coupled with inflammatory transcriptional programs. NF-κB activation, interferon-driven JAK/STAT signaling, and increased antigen presentation within cardiac tissue may collectively increase the visibility of cardiomyocytes to cytotoxic lymphocytes and intensify local damage (Wu et al., 2023; Michel et al., 2022; Subudhi et al., 2016; Lichtman, 2013; Huang et al., 2025). This framework also helps explain why ruxolitinib has attracted interest in steroid-refractory myocarditis: the drug targets a signaling axis that sits downstream of several inflammatory inputs rather than acting as a purely nonspecific immunosuppressant (Salem et al., 2023).

Metabolic state is likely another determinant of injury severity. Activated lymphocytes must sustain biosynthetic and energetic demands to persist, traffic, and execute cytotoxic programs, while inflamed cardiomyocytes face their own mitochondrial stress. Differences in metabolic fitness could therefore shape both the likelihood of immune infiltration and the tissue’s ability to tolerate it. Although this aspect remains underdeveloped clinically, it fits naturally within a cellular pharmacology model of susceptibility and resistance (Gergely et al., 2024b; Waliany et al., 2021a; Li et al., 2023).

## Determinants of susceptibility

The uneven distribution of cardiotoxicity across ICI-treated populations suggests that susceptibility is governed by more than drug exposure alone. Combination therapy is the clearest clinical risk factor identified so far. Concurrent disruption of distinct checkpoint pathways removes complementary layers of immune control and is consistently associated with a higher incidence of myocarditis and other major cardiac events (Salem et al., 2018; Dolladille et al., 2021; Ball et al., 2019; Hu et al., 2019; Nielsen et al., 2024; Jo et al., 2024). In quantitative terms, a recent meta-analysis of 54 randomized controlled trials (n = 38,264) and 24 observational studies (n = 12, 561, 455) confirmed significantly elevated odds of myocarditis and pericardial disease with ICI use compared with other cancer therapies (Sharma et al., 2024). The previously reported mortality rate of ICI-related myocarditis was substantially higher with combination ICI therapy (66%) than with monotherapy (44%), and combination regimens were also associated with a higher incidence of severe (grade ≥3) myocarditis (Salem et al., 2018; Mahmood et al., 2018).

Host factors are also likely to matter. Emerging studies have implicated HLA background, overlap syndromes involving myositis or myasthenia gravis, clonal hematopoiesis, and occult autoimmune predisposition as contributors to risk, although the evidence base remains relatively small and heterogeneous (Becker, 2025; Jaiswal et al., 2017; Martinez-Calle et al., 2018). These observations are more hypothesis-generating than definitive, but they reinforce the idea that cardiotoxicity is shaped by pre-existing immune architecture.

The broader tumor ecosystem may further influence vulnerability. Tumor-derived inflammatory signals, treatment history, and the intensity of systemic immune activation could all affect whether a patient’s cardiac tolerance mechanisms remain adequate after checkpoint blockade. Tumor types with high mutational burden, such as melanoma, generate a broader neoantigen repertoire that may increase the pool of activated T-cell clones capable of cross-reacting with cardiac antigens. Consistent with this, melanoma patients receiving combination ICI therapy show disproportionately high rates of myocarditis compared to other tumor types (Tang et al., 2025). In addition, tumors with high PD-L1 expression may influence the degree of systemic immune activation upon PD-1 blockade, potentially amplifying off-target effects. A recent scoping review of 29 studies confirmed that combination ICI regimens, ipilimumab use, and melanoma as the primary tumor type are among the most consistently reported risk factors for ICI-related cardiotoxicity (Wang et al., 2025). Direct mechanistic evidence linking specific tumor-derived factors to cardiotoxicity risk remains limited, and this represents a key Frontier where mechanistic oncology and cardio-immunology need to be integrated.

In practical terms, susceptibility probably emerges from layered risk: checkpoint combination, immune repertoire, baseline inflammatory state, and organ-specific reserve. The field still lacks a validated model that meaningfully combines these variables, which is why risk stratification remains much less precise than clinicians would like.

Toward an Integrative Risk Stratification Framework. Although no validated, ICI-cardiotoxicity-specific risk model currently exists, existing frameworks and recent data provide a foundation for a preliminary multi-parametric approach. The 2022 ESC cardio-oncology guidelines recommend baseline cardiovascular risk assessment before ICI initiation, including ECG, echocardiography, and cardiac biomarkers (Curigliano et al., 2020). The HFA-ICOS risk assessment tool, recently validated in a real-world cohort of 1,066 patients, demonstrated significant correlations between risk categories and the incidence of symptomatic cardiac events (Rivero-Santana et al., 2025). In parallel, a machine-learning-based prediction model using XGBoost achieved an area under the curve of 0.83 for predicting 30-day cardiotoxicity, with cardiac troponin T emerging as the most important predictor variable (Jialian et al., 2025). Building on these efforts, we propose that a preliminary integrative framework should incorporate three parameter domains: (1) clinical parameters, including ICI regimen type (combination versus monotherapy), pre-existing cardiovascular risk factors, autoimmune comorbidities, prior cardiotoxic therapy exposure, and age; (2) immunological parameters, including baseline inflammatory markers (C-reactive protein, interleukin-6), presence of pre-existing autoantibodies, HLA genotype where available, and history of other immune-related adverse events; and (3) molecular and biomarker parameters, including baseline high-sensitivity troponin, NT-proBNP, and potentially future markers such as T-cell receptor clonality signatures and circulating cytokine profiles. Such a framework remains conceptual and requires prospective validation in multi-center studies, but it provides a structured foundation for future risk-adapted monitoring strategies (Sharma et al., 2024) (Table 2).

**TABLE 2:** Biomarker domains relevant to early detection and risk stratification.

Biomarker domain	Examples	Current value	Clinical timing	Key limitations	Standardization challenges
Biochemical injury markers	hs-cTnI/hs-cTnT, BNP, NT-proBNP	Useful for surveillance and dynamic monitoring	Baseline; early cycles; acute workup; follow-up	Limited specificity for mechanism or subtype	Well standardized with clinical thresholds; ICI-specific cutoffs for subclinical cardiotoxicity remain undefined
Functional and imaging readouts	ECG, strain imaging, cardiac MRI	Improve phenotyping and help define severity	Baseline; symptom/biomarker-triggered; response monitoring	Often capture injury after biological onset	GLS requires vendor-specific normal ranges; cardiac MRI T1/T2 mapping is field-strength dependent (1.5 T vs. 3 T)
Immune-state markers	Cytokine profiles, T-cell subsets, TCR repertoire	Potentially closer to mechanism and susceptibility	Baseline; acute workup; response tracking	Not standardized; many signals are context dependent	Cytokine profiling lacks consensus reference ranges; results vary with assay platform and sample timing
Emerging molecular markers	microRNAs, extracellular vesicles, advanced PET tracers	May support earlier or more specific detection	Biobanking; early sampling; longitudinal validation	Require validation in prospective cohorts	MicroRNA quantification varies across platforms (qPCR vs. sequencing); EVs lack standardized isolation protocols

## Therapeutic resistance and mechanism-based intervention

Current management of ICI myocarditis remains anchored to high-dose corticosteroids, and that approach is appropriate for early disease control. Yet steroid responsiveness is far from uniform. A subset of patients stabilizes rapidly, whereas others continue to worsen despite prompt treatment, indicating that resistance to first-line immunosuppression is clinically consequential (Brahmer et al., 2018; Salem et al., 2023; Thompson et al., 2022; Schneider et al., 2021; Lyon et al., 2022; Zhang et al., 2020b). In this review, we define “therapeutic resistance” in the context of ICI cardiotoxicity as the failure of standard immunosuppressive therapy (primarily high-dose corticosteroids at ≥1 mg/kg methylprednisolone equivalent) to achieve hemodynamic stabilization or meaningful reduction in inflammatory markers within 48–72 h of treatment initiation, necessitating escalation to second-line agents. This can be further subdivided into primary resistance (minimal or no initial response to corticosteroids) and secondary resistance (initial improvement followed by clinical deterioration upon steroid taper). Recent cohort data show that approximately one-third of ICI myocarditis patients develop major adverse cardiac events despite treatment, and patients requiring three or more immunosuppressive agents have significantly higher mortality (Ozaki et al., 2025) (Table 3).

**TABLE 3:** Mechanism-based therapeutic strategies and their translational rationale.

Intervention	Proposed target	Clinical position	Key sources	Evidence level
High-dose corticosteroids	Broad suppression of inflammatory signaling and lymphocyte activation	Current first-line therapy, but response remains heterogeneous	Brahmer et al. (2018), Schneider et al. (2021), Lyon et al. (2022), Zhang et al. (2020b), Salem et al. (2019),	Expert consensus (ESMO, ASCO); response rate 50%–70% initial stabilization
Abatacept	CD28-B7 costimulatory axis	Most developed mechanism-based rescue strategy	Salem et al. (2023), Tay et al. (2017),	Case series (Salem et al., 2019, n = 4; expanded cohort); clinical improvement in steroid-refractory cases
Ruxolitinib	JAK1/2-mediated inflammatory amplification	Used in severe or steroid-refractory disease, often with abatacept	Salem et al. (2023),	Case series; used in combination with abatacept in severe or refractory disease
Anti-thymocyte globulin	T-cell depletion	Reserved for refractory myocarditis in small series	Zhang et al. (2021)	Individual case reports (n = 2–3 per series)
Infliximab/IVIG/other second-line approaches	Context-dependent inflammatory pathways	Used selectively; evidence base remains limited	Esfahani et al. (2019), Frigeri et al. (2018), Arangalage et al. (2017), Isogai et al. (2015), Murtagh et al. (2024),	Individual case reports and small case series; evidence base limited

Mechanism-based rescue strategies have begun to change the therapeutic conversation. Abatacept, by interfering with CD28–B7 costimulation, addresses a core activation pathway in T cells. Ruxolitinib targets downstream inflammatory signaling, particularly within interferon-linked circuits. The combination has produced striking results in selected clinical series and is conceptually appealing because it matches therapeutic intervention to the biology of the syndrome more closely than empiric escalation alone (Salem et al., 2023; Salem et al., 2019).

Other second-line approaches, including anti-thymocyte globulin, infliximab, intravenous immunoglobulin, and additional immunosuppressive agents, remain supported mainly by case series and small cohorts (Tay et al., 2017; Zhang et al., 2021; Esfahani et al., 2019; Frigeri et al., 2018; Arangalage et al., 2017; Isogai et al., 2015). Their continued use reflects clinical necessity, but it also highlights a deeper problem: ICI-associated cardiotoxicity is still being treated as if it were a single disease. If multiple mechanistic subtypes exist, then variable therapeutic responses should be expected rather than surprising.

The unresolved question is how to mitigate toxicity without fully erasing antitumor benefit. That challenge is central to the special topic of sensitivity and resistance. A successful toxicity-directed strategy will likely need to preserve enough systemic immune activity to maintain tumor control while selectively interrupting tissue-destructive circuits in the heart. The abatacept–ruxolitinib combination is particularly instructive in this regard, because it targets specific T-cell activation and inflammatory amplification pathways rather than imposing broad immunosuppression. Observational data suggest that the occurrence of immune-related adverse events, including cardiotoxicity, may be associated with improved antitumor efficacy (Zhou et al., 2020), which underscores the complexity of this balance. The concept of “permissive cardiotoxicity,” recently discussed by the JACC CardioOncology Expert Panel, involves individualized risk–benefit assessments for continuing ICI therapy in patients with mild cardiac events, though the supporting evidence base remains limited to case series (Raisi-Estabragh et al., 2024). Clinical trials specifically designed to assess selective immunosuppression strategies in the context of ICI cardiotoxicity are urgently needed.

## Biomarkers and translational readouts

Early detection remains difficult because no single biomarker captures the full biology of ICI-related cardiotoxicity. To provide a structured translational roadmap, available biomarkers can be stratified into three tiers based on their current clinical maturity. Tier 1 (clinically established) markers include high-sensitivity troponins (hs-cTnI/hs-cTnT) and natriuretic peptides (BNP, NT-proBNP). High-sensitivity troponins remain the most widely used markers and are clinically valuable, especially when interpreted dynamically against baseline values. Natriuretic peptides, electrocardiographic changes, and echocardiographic strain measurements add useful contextual information but still lack the specificity needed for confident mechanistic classification (Mahmood et al., 2018; Murtagh et al., 2024; Waliany et al., 2021b; Spallarossa et al., 2019; Lehmann et al., 2023; Vasbinder et al., 2022; Awadalla et al., 2020; Thavendiranathan et al., 2021). Recent retrospective data demonstrate that troponin can predict major adverse cardiac events in ICI myocarditis patients with an area under the curve of 0.82 (Ozaki et al., 2025).

Tier 2 (approaching clinical readiness) markers include cardiac magnetic resonance imaging with T1 and T2 mapping and global longitudinal strain (GLS) echocardiography. These modalities offer tissue-level characterization and can detect subclinical myocardial inflammation before overt hemodynamic compromise, though they require specialized expertise and currently lack universally accepted cutoff values specific to ICI-related cardiotoxicity (Awadalla et al., 2020; Thavendiranathan et al., 2021). Tier three (investigational) markers encompass cytokine profiles, microRNA signatures, extracellular vesicle content, and advanced imaging tracers such as ^86^Ga-FAPI (Finke et al., 2021; Pudil et al., 2020; Xu et al., 2025; Lim et al., 2019; Michel et al., 2019; Xia et al., 2020). These approaches show promise for earlier or more mechanism-specific detection but require prospective validation in adequately powered cohorts and standardization of assay methodology across centers. At the same time, large observational cohorts underscore how much signal may be missed when surveillance is inconsistent or driven only by symptoms (Vasbinder et al., 2022; Laenens et al., 2022; D'Souza et al., 2021; Bonaca et al., 2019).

The main limitation of the current biomarker literature is not the absence of candidates but the absence of a coherent framework. Most studies evaluate biomarkers one at a time, whereas the clinical syndrome is heterogeneous and probably biologically composite. A more useful strategy would combine biochemical injury markers, immune-state measurements, and imaging-based evidence of tissue involvement to identify mechanistic subsets rather than merely flagging injury after it has already become established. Standardization challenges remain significant: cytokine profiling lacks consensus reference ranges and cutoff values, microRNA quantification varies substantially across platforms, and cardiac MRI T1/T2 mapping requires field-strength-specific reference values that may differ between vendors.

**FIGURE 2:**
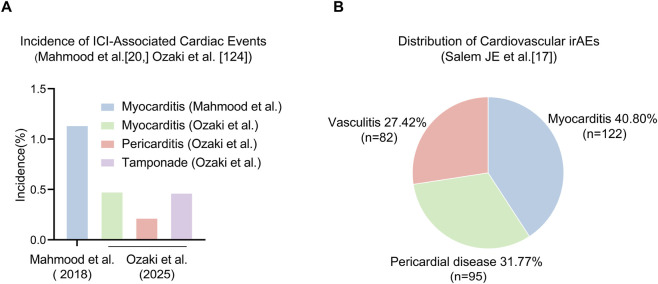
Clinical landscape of immune checkpoint inhibitor-associated cardiotoxicity **(A)** Incidence of selected ICI-associated cardiac events reported in two independent cohorts. Mahmood et al. reported myocarditis from a multicenter registry of 35 cases with 105 ICI-treated controls, whereas Ozaki et al. analyzed myocarditis and pericardial diseases in 88,928 ICI-treated cancer patients from the real-world TriNetX network. Because these studies differed in design, data source, patient population, case definition, and follow-up window, the incidence values are descriptive and should not be interpreted as direct head-to-head comparisons **(B)** Distribution of major cardiovascular immune-related adverse events reported by Salem et al. in a retrospective pharmacovigilance analysis.

From a translational standpoint, biomarker development should serve two goals. The first is earlier recognition of clinically significant myocarditis. The second, which is arguably more important for the field, is identification of patients whose biology places them near the threshold of toxicity before irreversible injury occurs. To place these mechanistic considerations into clinical context, Figure 2 summarizes the reported incidence of cardiotoxic events across ICI regimens and the relative distribution of major cardiac toxicities.

## Clinical, translational, and nursing implications

Although cardiotoxicity is often discussed as an adverse effect, it may be more accurate to view it as one manifestation of systemic immune modulation. This perspective matters because it links toxicity, antitumor efficacy, and host susceptibility instead of treating them as independent phenomena. It also explains why some clinical observations appear contradictory: the same immune processes that improve tumor control may, in a subset of patients, destabilize tissue-specific tolerance in the heart (Zhou et al., 2020).

Clinically, this means that the optimal response is unlikely to be one-size-fits-all escalation of immunosuppression. Better integration between oncology, cardiology, and translational immunology is needed, not only for acute management but also for trial design, biospecimen collection, and mechanistic subgrouping (Curigliano et al., 2020; Herrmann, 2020; Chinese Society of Clinical Oncology CSCO, 2023; Neilan et al., 2018; Balanescu et al., 2020; Zhou et al., 2019; Stein-Merlob et al., 2021; Totzeck et al., 2019; Lancellotti et al., 2019; Hayek et al., 2019). Digital monitoring tools may eventually support follow-up and surveillance, but technology will only be useful if it is embedded within biologically informed clinical pathways (Steinhubl et al., 2015; Perez et al., 2019; Hannun et al., 2019). On the basis of the 2022 ESC cardio-oncology guidelines and the HFA-ICOS framework, a practical monitoring algorithm would include: (1) baseline cardiovascular assessment before ICI initiation, encompassing a 12-lead ECG, echocardiography with GLS measurement, and baseline hs-cTnI/T and BNP/NT-proBNP; (2) serial troponin monitoring at each treatment cycle for at least the first 3 months, with ECG repeated at clinically determined intervals; (3) risk-adapted surveillance intensity, with high-risk patients (combination ICI, pre-existing cardiovascular disease, autoimmune comorbidities) receiving more frequent monitoring; and (4) a stepwise escalation pathway from clinical suspicion through urgent echocardiography, cardiac MRI with T1/T2 mapping, and, when indicated, endomyocardial biopsy, with parallel initiation of high-dose corticosteroids when myocarditis is suspected (Curigliano et al., 2020; Rivero-Santana et al., 2025; Raisi-Estabragh et al., 2024).

At the bedside, these translational considerations still depend on consistent clinical surveillance. Within the multidisciplinary framework spanning oncology, cardiology, intensive care, pharmacy, and nursing, structured nursing assessment remains particularly important because ICI cardiotoxicity may first present with subtle symptoms such as new fatigue, palpitations, mild dyspnea, or isolated biomarker changes. Standardized symptom screening at each treatment visit, careful collection of serial biomarker samples, electrocardiographic quality assurance, patient education on warning symptoms, and rapid escalation pathways can strengthen early recognition and continuity of care (Thompson et al., 2022; Schneider et al., 2021; Lyon et al., 2022; Curigliano et al., 2020; Herrmann, 2020; Chinese Society of Clinical Oncology CSCO, 2023; Neilan et al., 2018; Balanescu et al., 2020; Zhou et al., 2019; Steinhubl et al., 2015; Perez et al., 2019; Hannun et al., 2019). As mechanism-based therapies become more common, nursing teams will also need familiarity with the monitoring requirements and toxicity profiles of newer immunosuppressive strategies.

The field is now well positioned to move from descriptive recognition to selective intervention. That transition will require studies that link immune profiling, tissue biology, and therapeutic response in the same patients. Without that integration, sensitivity and resistance will remain descriptive labels rather than actionable concepts.

## Conclusion

ICI-associated cardiotoxicity sits at the interface of cancer pharmacology, tissue-specific immunology, and host susceptibility. The field has moved beyond seeing myocarditis as a rare idiosyncratic complication, yet the biology remains incompletely resolved. Current evidence supports a model in which checkpoint blockade disrupts cardiac immune homeostasis, releases or amplifies pathogenic T-cell programs, and engages innate and stromal circuits that sustain injury (Axelrod et al., 2022; Munir et al., 2026; Salem et al., 2023; Wei et al., 2021; Rubio-Infante et al., 2022; Gong et al., 2023; Gergely et al., 2024b; Wu et al., 2023; Varricchi et al., 2017; Michel et al., 2022; Horiguchi et al., 2023; Waliany et al., 2021a; Li et al., 2023; Quagliariello et al., 2020).

The next phase of progress depends on moving from recognition to discrimination. Patients do not all develop toxicity through identical pathways, and they should not be expected to respond identically to treatment. Mechanistically informed biomarkers, careful risk stratification, and selective toxicity-directed therapies will be necessary if the field is to reduce cardiac injury without simply neutralizing the therapeutic promise of immune checkpoint blockade.

Seen in that light, cardiotoxicity is not only a complication of immunotherapy. It is also a revealing model of how sensitivity and resistance operate across the tumor–host ecosystem.
